# Ultra-Processed Food Consumption and Relation with Diet Quality and Mediterranean Diet in Southern Italy

**DOI:** 10.3390/ijerph191811360

**Published:** 2022-09-09

**Authors:** Justyna Godos, Francesca Giampieri, Wahidah H. Al-Qahtani, Francesca Scazzina, Marialaura Bonaccio, Giuseppe Grosso

**Affiliations:** 1 Department of Biomedical and Biotechnological Sciences, University of Catania, 95123 Catania, Italy; 2 Research Group on Food, Nutritional Biochemistry and Health, European University of the Atlantic, 39011 Santander, Spain; 3 Department of Food Sciences & Nutrition, College of Food & Agriculture Sciences, King Saud University, Riyadh 11451, Saudi Arabia; 4 Department of Food and Drug, University of Parma, Via Volturno 39, 43125 Parma, Italy; 5 Department of Epidemiology and Prevention, IRCCS NEUROMED, 86077 Pozzilli, Italy

**Keywords:** ultra-processed foods, NOVA classification, diet quality, macronutrients, micronutrients, vitamins, minerals, Mediterranean diet

## Abstract

Ultra-processed food (UPF) consumption has been the focus of major attention due to their potential effects on human health. The aim of this study was to investigate the intake of UPFs in a sample of southern Italian individuals and assess its relationship with nutrient profile and dietary quality parameters. A cross-sectional study was conducted on 1936 individuals older than 18 years randomly selected from the general population. A total of 110 food times have been categorized based on the level of processing using the NOVA classification. The average daily energy intake of the sample was 2091.1 kcal, 38.7% of which were from the NOVA group of unprocessed/minimally processed foods, 5.7% from processed culinary ingredients, 38.3% from processed foods, and 17.9% from the UPFs group. UPFs were more consumed among young, unmarried individuals, with high cultural level, smokers, and often eating out of home. The mean energy share of UPFs varied from 6.3% of total daily energy intake for individuals in the lowest quintile of UPF consumption to 34.2% for those in the upper quintile. Within the UPF group, the highest energy contribution was provided by fast foods and sweets. Compared to the lowest quintile of UPF consumption, individuals in the highest quintile consumed, on average, additional 300 kcals per day and less fiber. Some plant-derived vitamins, such as vitamin A and vitamin C showed an inverse trend toward increasing shares of UPF consumption, while sodium intake increased. A significant higher intake of UPFs in individuals meeting the European and Italian dietary recommendations for carbohydrates, vitamin B12, vitamin D, and vitamin E was found, while UPFs were less consumed among those meeting the recommendations for total fats, fiber, sodium, potassium, and vitamin C. Finally, individuals displaying a “healthier” dietary profile, such as higher adherence to either the Mediterranean diet, the Dietary Approaches to Stop Hypertension, the Alternate Diet Quality Index, and the Diet Quality Index-International, consumed less UPFs and more unprocessed/minimally processed foods, with minor variation in the other NOVA food categories. In conclusion, consumption of UPF in southern Italy is in line with those reported in some other Mediterranean countries, although it negatively impacted the nutrient profile. It is important to monitor the consumption of UPFs before their availability and popularity put the grounds on younger generations’ dietary habits.

## 1. Introduction

Traditional dietary patterns based on the consumption of unprocessed or minimally processed foods, such as those typical of the Mediterranean area, are strongly associated with a lower risk for the onset and development of several non-communicable diseases (NCDs), such as obesity, types 2 diabetes, cardiovascular diseases and some types of cancer [1,2], which represent the main causes of disability and premature death worldwide [3,4]. In recent years, rising evidence has shown that, besides nutritional composition, the industrial food processing may exert a negative role on human health [5,6,7]. Since the volume and the consumption of these industrially processed foods have dramatically increased everywhere, understanding their impact on human health has become of primary importance. To this purpose, several methods to classify food processing have been conceived recently, among which the most renowned is NOVA classification. This system categorizes foods according to the nature, the extent and the purpose of the industrial food processing into four groups: (i) unprocessed or minimally processed foods, including edible parts of plants, animals, algae and fungi that usually undergo industrial processes able to preserve their nature (i.e., grinding, fractioning, drying, crushing, boiling, filtering, pasteurization, roasting, chilling, freezing and refrigeration), (ii) processed culinary ingredients, such as sugar, butter, salts and oils, derived from nature or from Group 1 foods and subjected to industrial processes that increase their durability (i.e., drying, refining, pressing, grinding and milling), (iii) processed foods, including freshly made breads, bottled vegetables, cheese, canned fish or fruits in syrup, in which some substances, such as salt, sugar or oils, are added to enhance their sensorial characteristics or their durability and (iv) ultra-processed foods (UPF), that are formulations made, in part or completely, from substances derived from foods and additives and subjected to a multitude of industrial processes [8,9,10]. UPF are calorie-dense foods, usually characterized by high levels of added sugars, salt, oils, fats and other substances extracted from foods, such as gluten, whey, lactose and casein, or derived from further food processing, including maltodextrin, soy protein isolate, hydrolyzed proteins, interesterified or hydrogenated oils, high-fructose corn syrup and invert sugar [8,9,10]; other common substances found in UPF are additives, such as stabilizers, antioxidants, preservatives, colors, non-sugar sweeteners, flavor enhancers, emulsifiers, humectants and sequestrants [8,9,10]. Consequently, UPF are hyper-palatable, imperishable, ready-to-consume, easily accessible and cheap products, which are successfully displacing the use of unprocessed or minimally processed foods worldwide, also thanks to the massive marketing strategies and social campaigns undertaken.

UPFs have been suggested to impact the overall diet quality of populations globally [11]. Due to the nature of their ingredients, UPFs represent an unhealthy dietary alternative growingly preferred among the younger generations [10]. Their consumption has been indeed associated with a general worsening of the diet quality [12,13,14,15,16], as well as with a higher risk to develop different chronic NCDs, including obesity [17,18,19], metabolic syndrome [20], gastrointestinal diseases [21], cardiovascular diseases [22,23,24] and some types of cancer [25], among others. Unsurprisingly, the annual average growth of UPF sales is constantly increasing, especially in the middle-income countries, where the sale rate is estimated around 10% per year [26,27]. Additionally, UPF consumption is extraordinarily high in non-Mediterranean countries, such as USA [28], UK [29], Australia [30] and Canada [31], while it seems to be moderate in Mediterranean countries, such as Spain, where the long-traditional diet is still prevalent [32].

Similarly to other countries of the Mediterranean area, the nutritional and lifestyle habits in Italy are gradually shifting from the traditional Mediterranean diet to a more “Westernized” dietary pattern, with consequent adverse health outcomes, especially in younger people [33,34,35,36]. Despite this, only a limited number of studies exploring UPF consumption in the Italian population have been published so far [23,37], none of which providing detailed information regarding food subgroups and the relation with diet quality parameters. The aim of this work was to describe the pattern of consumption of UPFs and its relationship with the nutrient profile and dietary quality in Italian adults living in Sicily, southern Italy.

## 2. Materials and Methods

### 2.1. Study Population

The Mediterranean healthy Eating, Aging, and Lifestyle (MEAL) study is an observational study aimed to evaluate the correlation between Mediterranean dietary and lifestyle habits and non-communicable diseases. The protocol of this study is illustrated in detail in the paper of [38]. In brief, a sample of 2044 men and women aged 18 or more years old, randomly enrolled between 2014 and 2015 in the main districts of Catania, a city of southern Italy, was included in the baseline data, by using the list of registered records of local general practitioners. Data were stratified by 10-year age groups and sex. In order to provide a specific relative precision of 5% (Type I error, 0.05; Type II error, 0.10), considering an anticipated 70% participation rate, the theoretical sample size was estimated to 1500 individuals.

In detail, 2405 subjects were invited to participate in the study, 2044 participants accepted (response rate of 85%), while 361 individuals refused. The aims of the study were carefully explained to all participants, who gave their written informed consent. All the procedures were performed in agreement with the Declaration of Helsinki (1989) of the World Medical Association. The study protocol has been revised and authorized by the concerning ethical committee.

### 2.2. Data Collection

In order to visualize the response options, a paper copy of the questionnaire was provided to all participants during face-to-face assisted personal interviews. Tablet computers were used by the interviewer to directly register the final answers. Information concerning sociodemographic factors and lifestyle habits was recorded. The sociodemographic data comprised sex, age at recruitment and educational status [low (primary/secondary school), medium (high school), and high (university)], while lifestyle variables included smoking status (non-smoker, ex-smoker, and current smoker), physical activity [low, moderate, high; determined by using the International Physical Activity Questionnaire (IPAQ) [39], as well as alcohol drinking [none, moderate drinker (0.1–12 g/d) and regular drinker (>12 g/d)].

### 2.3. Dietary Assessment

Aiming to determine the dietary intake, two food frequency questionnaires (FFQ, a long and a short version) previously proved for validity and reliability for the Sicilian population were adapted [40,41]. For the purposes of this study, only data from the long version were imputed, consisting of a 110-item FFQ. Employing the determination of the food intake, the energy content as well as the macro- and micro-nutrients intake was calculated through a comparison with food composition tables of Council for Research in Agriculture and Analysis of Agricultural Economy (CREA) [42]. Intake of seasonal foods referred to consumption during the period in which the food was available and then adjusted by its proportional intake over one year.

### 2.4. Food Classification

Based on the NOVA classification, food items have been classified as follow: group 1, unprocessed or minimally processed foods (i.e., rice and other cereals, meat, fish, milk, eggs, fruit, vegetables, nuts, etc.); group 2, processed culinary ingredients (i.e., sugar, vegetable oils and butter); group 3, processed foods (i.e., processed breads and cheese); group 4, ultra-processed foods (i.e., confectioneries, salty snacks, fast-foods, soft drinks, etc.) [9]. Based on this classification, all 110 items of the long FFQ were included in the classification and used for the analyses.

### 2.5. Diet Quality Measures

Several variables have been assessed in order to investigate the relation between UPF consumption and diet quality. Specifically, the proportion of UPF in the diet was related to various dietary patterns and scores, including the Mediterranean diet, the Dietary Approaches to Stop Hypertension (DASH), the Alternate Diet Quality Index (AHEI), and the Diet Quality Index-International (DQI-I). Adherence to the Mediterranean dietary pattern has been assessed through a literature-based scoring system [43], which provides a positive scoring when reporting consumption of food groups in line with the Mediterranean diet (i.e., fruits and vegetables, cereals, legumes, fish, olive oil, moderate alcohol consumption) and negative scoring related to consumption of food groups not in line (i.e., meat and dairy products, excess or no alcohol intake). The nine food groups contribute to build a score score ranging from 0 point (lowest adherence) to 18 points (highest adherence). For the purposes of this study, participants were categorized in 3 groups indicating low (<7 points), medium (7–12 points), and high (>12 points) level of adherence to the Mediterranean diet [44]. The DASH diet is a score composed by 9 points calculated based on (i) the consumption of recommended daily servings of six food groups (whole grains, fruits, vegetables, meat and dairy products, nuts/seeds/legumes), (ii) average daily consumption of saturated fat less than the recommended 5% of total energy intake, (iii) average daily consumption of added sugar intake <3% of total energy intake, and (iv) alcohol intake equal or below the recommended 2 drinks/d for men and 1 drink/d for women. Participants were finally categorized in 3 groups indicating low (<4 points), medium 4–5 points), and high (>5 points) adherence to the DASH diet [45]. The A-HEI was calculated based on seven food/nutrient items (fruits and vegetables, cereal fiber, nuts, ratio of white to red meat, trans fats, alcohol, ratio of polyunsaturated to saturated fatty acids) each contributing 0 to 10 points indicating no to fully meeting the nutritional recommendation (and intermediate scores proportionally indicating intermediate intakes) and use of a multivitamin contributing either 7.5 or 2.5 points for use/no use, respectively. The total A-HEI score ranges from 2.5 (worst) to 87.5 (best), with participants categorized in 3 groups indicating low (<35 points), medium (35–41 points), and high (>41 points) adherence to the score [46]. Finally, the DQI-I was calculated based on food/nutrient items considering (i) variety of consumption of five food groups (fruits and vegetables, meat/poultry/fish/egg, dairy/beans, and grains); (ii) adequacy, considering minimum adequate daily intake for fruits and vegetables, grains, and fiber (dependent on daily energy intake), a proportion of >10% of total energy from protein, and selected cut-off values derived from the recommended daily intakes for Italian adults for iron, calcium, and vitamin C intake; (iii) moderation, considering maximum intake for foods and nutrients that may need restriction; (iv) overall balance, considering the proportion of energy sources and fatty acid composition. The final score ranges from 0 to 100 points, with participants categorized in 3 groups indicating low (<41 points), medium (41–47 points), and high (>47 points) adherence to the DQI-I [47].

Finally, the agreement with dietary recommendations of the European proposed values for macronutrient intake of the European Food Safety Agency (EFSA) [48] and those proposed by the Italian Society of Human Nutrition “*Livelli di Assunzione di Riferimento di Nutrienti*” (LARN) [49] for selected nutrients has been further investigated.

### 2.6. Statistical Analysis

The mean share of all NOVA food group and subgroups to the total daily energy intake was estimated. The participants were categorized into quintiles of energy shares of UPFs. The energy share of each NOVA food group and subgroup was estimated across quintiles of UPF consumption and differences between groups were tested with Student’s *t*-test and ANOVA for paired and multiple group comparisons after testing for normality of distribution of the variables. Test for linearity of differences was performed through contrasts analysis. The distribution of the mean nutrient intake across quintiles of energy shares of UPFs was also calculated and differences between groups tested. The mean intake of the four NOVA food group by level of adherence to the Mediterranean diet and adherence to dietary recommendations were finally calculated and differences between groups tested. All reported *p* values were based on two-sided tests and compared to a significance level of 5%. SPSS 25 (SPSS Inc., Chicago, IL, USA) software was used for all the statistical calculations.

## 3. Results

**Figure 1:**
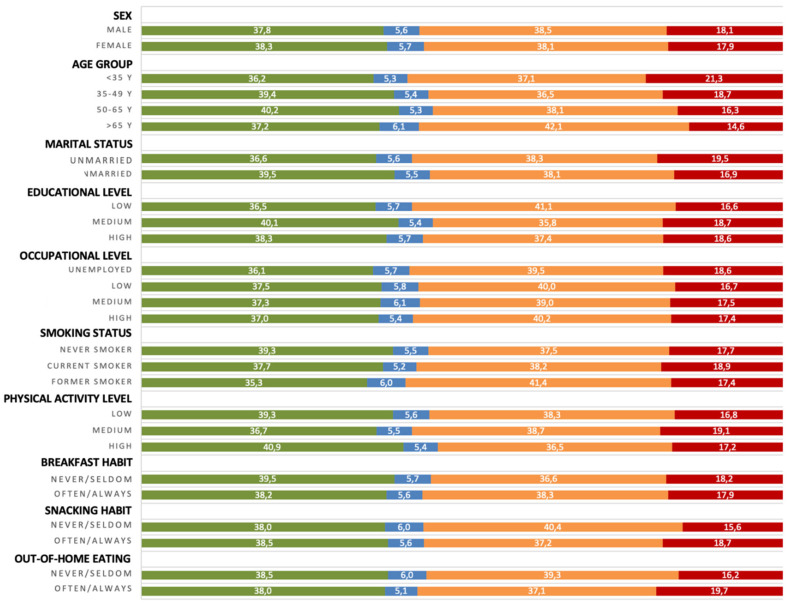
Percent distribution of food group consumption by level of processing across background variables. Green color stands for unprocessed/minimally processed foods; blue color stands for processed culinary ingredients; brown color stands for processed foods; red color stands for ultra-processed foods.

The average daily energy intake of the sample was 2091.1 kcal, 38.7% of which were from the NOVA group of unprocessed/minimally processed foods, 5.7% from processed culinary ingredients, 38.3% from processed foods, and 17.9% from the UPFs group. An overview of average consumption of foods by processing level is presented in Figure 1, with detailed data reported in Supplementary Table S1. The largest variation in energy shares of UPFs among background characteristics has been observed across age groups, being UPFs more consumed among young individuals and linearly decreasing for increasing categories of age groups (Figure 1). Among other background characteristics showing differences in UPF intake, individuals unmarried (*p* < 0.001), current smokers (*p* = 0.046) with higher cultural level (*p* < 0.001) and medium physical activity level (*p* < 0.001) reported consuming more UPFs (Figure 1). UPF consumption did not substantially differed by sex and occupational level (Figure 1). Among eating habits, individuals having often snacks (*p* = 0.001) and out-of-home eating (*p* < 0.001) reported higher consumption of UPFs, while there were no differences for breakfast habits (Figure 1).

Among the main food subgroups characterizing the NOVA categories, grains and pasta, fruits, vegetables, and legumes accounted for most of the energy of the unprocessed/minimally processed foods (Table 1). Among processed culinary ingredients, vegetable oils provided the highest energy contribution (Table 1). Within processed foods, most of the energy came from bread and cheese (Table 1). Within the UPF group, the highest energy contribution was provided by fast foods and sweets (biscuits, pastries, cakes, ice creams, confectionery and creams) (Table 1).

The mean energy share of UPFs varied from 6.3% of total daily energy intake for individuals in the lowest quintile of UPF consumption to 34.2% for those in the upper quintile (Table 2). All specific foods within the UPF NOVA group increased linearly across quintiles of UPF consumption, with the largest variation observed for breakfast cereals, confectionery and creams, salty snacks, carbonated soft-drinks, and confectioned juices, which were rarely consumed in the lowest quintile groups (Table 2). An opposite trend was observed for the unprocessed/minimally processed food group, despite there were no significant decreasing linear trends for individual subgroups of foods, except for fruits (Table 2). An additional analysis has been conducted by age groups (Supplementary Table S2). Interestingly, despite the mean UPF intake is higher in younger than older participants, the extreme categories were not substantially different (in the younger group, the mean intake in the highest quintile of UPFs was even smaller than in the older one) with no large differences in the trends observed for the whole sample, with some difference of consumption of certain foods in the lowest quintile of UPF consumption between younger and older participants (i.e., younger individuals ate slightly more fish, vegetables, fruit, nuts, fast foods than older ones).

The comparison of mean intake of total energy intake and nutrients across energy shares of quintiles of UPF consumption is reported in Table 3. Compared to the lowest quintile of UPF consumption, individuals in the highest quintile consumed, on average, additional 300 kcals per day; significant increasing trends toward higher consumption of UPFs were observed for all macronutrients, with exception of specific analysis of n-3 polyunsaturated fatty acids (either from plant or seafood sources), and for carbohydrates (Table 3). In contrast, an inverse trend for fiber intake was found, ranging from 38 to 31 g/d between the first and the fifth quintile of UPF consumption. Concerning micronutrients, some plant-derived vitamins, such as vitamin A and vitamin C showed an inverse trend toward increasing shares of UPF consumption, while others, such as vitamin E, vitamin D, and vitamin B12 showed a linear increasing trend; also mineral intake was significantly differently distributed across quintiles of dietary shares of UPF consumption, although sodium intake resulted in an increased trend toward higher intakes of UPFs, while potassium intake tended to decrease, although not with a clear trend over categories (Table 3). When running again the same analysis by age groups, the observed trends were not identical than in the whole cohort, being the intake of n-3 PUFA, carbohydrates, potassium, vitamin A, vitamin E, vitamin D, vitamin B12 lower in higher consumers of UPFs compared to lower ones among younger individuals while opposite trends were reported in the older age group (Supplementary Table S3).

**Figure 2:**
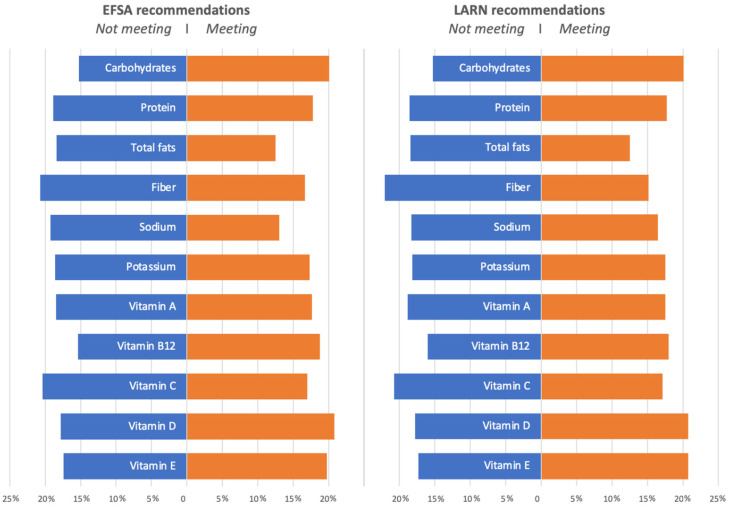
Percentage of UPF consumption in individuals meeting and not meeting the European and Italian nutritional recommendations for selected macro- and micro-nutrients.

Consistently, when exploring the mean consumption of UPFs between individuals meeting the European and Italian dietary recommendations and those who did not, there was a significant higher intake in those meeting the recommendations for carbohydrates (*p* < 0.001), vitamin B12 (*p* < 0.001 for EFSA but not significant for LARN), vitamin D (*p* = 0.003), and vitamin E (*p* < 0.001), while UPFs were less consumed among those meeting the recommendations for vitamin A (*p* = 0.009 for LARN but not significant for EFSA) total fats (*p* < 0.001), fiber (*p* < 0.001), sodium (*p* < 0.001 for EFSA and *p* = 0.002 for LARN), potassium (*p* = 0.007 for EFSA but not significant for LARN), and vitamin C (*p* < 0.001) for both EFSA and LARN (Figure 2). Detailed data are reported in Supplementary Table S4.

**Figure 3:**
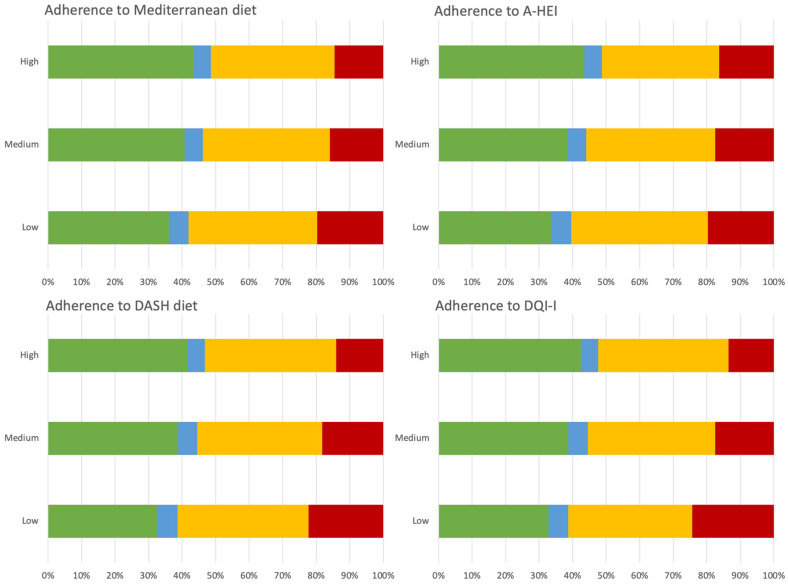
Percent distribution of food group consumption by level of processing across level of adherence to selected a-priori defined healthy dietary patterns. Green color stands for unprocessed/minimally processed foods; Blue color stands for processed culinary ingredients; yellow color stands for processed foods; red color stands for ultra-processed foods.

Finally, the energy shares of NOVA food groups by level of adherence to various dietary patterns and indices of diet quality is shown in Figure 3: irrespectively of the dietary score used, individuals displaying a “healthier” dietary profile (meaning higher adherence to either the Mediterranean diet, the DASH diet, the A-HEI, or the DQI-I) consumed less UPFs and more unprocessed/minimally processed foods, with minor variation in the other NOVA food categories (Figure 3). Detailed data are reported in Supplementary Table S5.

## 4. Discussion

This study aimed to describe the consumption of UPFs in a southern Italian cohort of adults and testing for its relationship with various parameters of diet quality. The average daily intake of UPFs registered in this cohort is in line with the rates reported in the Italian Nutrition & Health Survey (INHES), a study conducted on 9139 participants aged 5–97 years in which the energy share of UPFs was 17.3% [37]. These average consumption of UPFs are also consistent with those reported for other Mediterranean countries. For instance, the Study on Nutrition and Cardiovascular Risk in Spain (ENRICA) including 12,948 individuals, in which UPFs accounted for about 24% of daily energy intake [32], and another representative Spanish cohort from the Diet and Risk of Cardiovascular Diseases (CVD) in Spain (DRECE) study including 4679 individuals reported an average consumption of 24% of energy intake from UPFs [50]. In addition, data from the National Food, Nutrition and Physical Activity Survey (2015–2016) of the Portuguese population including 3852 participants showed an average daily intake of 22% of daily energy share from UPFs, ranging from 6.5% to 44.1% in the lowest and highest quintile of intake, respectively [51]. In contrast, findings from a French survey from the *Étude Nationale Nutrition Santé* (ENNS) conducted on 2642 participants, showed substantial higher intake of UPFs than other Mediterranean countries (average of 31.1%, ranging from 12.8% to 51.5% daily energy shares of UPFs in first and fifth quintiles, respectively) [52]. Moreover, studies from non-Mediterranean countries, such as Canada [12], UK [29], Australia [30], and US [53] showed substantial higher intakes of UPFs, with average intakes of more than 50% of total energy share in UPF reaching up to an average 80% in the highest quintiles of dietary shares of energy from UPFs. Compared to the aforementioned cohorts and samples, also the preferred consumption of food subgroups reported in the present study was substantially different from those conducted in countries outside the Mediterranean area: in fact, the results from this study showed a peculiar characterization of food subgroup consumption, with a trend toward higher intakes of pasta and carbohydrates among unprocessed/minimally processed foods, vegetables oils among processed culinary ingredients, bread among processed foods, and sweets among UPFs. This pattern of consumption is quite unique compared to the data from other samples worldwide, in which the consumption of pasta (i.e., about 1–2% in US vs. about 10% in Italy), fruits, vegetables, and legumes (i.e., less than 1% in US vs. about 5% in Italy) is reported to be generally much lower, while other food products, such as carbonated soft drinks (i.e., up to 7% in US vs. 0.6% in Italy), salty snacks (i.e., almost 5% in US vs. less than 1% in Italy), and breakfast cereals (i.e., 3% in US vs. less than 1% in Italy) among others, is generally higher [53]. Interestingly, we also noted that in the present study the intake of UPFs was much higher in the highest quintile than in all other groups; this may reflect a certain preference for UPFs by a subgroup of the sample that is likely to consume this type of food not just in higher quantity, but probably as part of a dietary pattern poorer in other types of foods (i.e., less processed ones).

A recent meta-analysis pooling together nutritional data from nationally-representative samples worldwide showed a global trend toward a poorer nutritional quality correlated with higher consumption of UPFs (increase in free sugars, total fats, and saturated fats, as well as a decrease in fiber, protein, potassium, zinc, and magnesium, and vitamins A, C, D, E, B12, and niacin) [11]. The results of the present study are not completely in line with those previously reported, since the consumption of only a few vitamins and sodium was found to be negatively affected by higher intake of UPFs. This may reflect the overall scarce consumption of UPFs in this cohort compared to US, Canadian, and Australian cohorts, with the main contributors being sweet foods rather than other commercially relevant UPFs, which are, in fact, not much consumed in southern Italy [33,54]. It may be possible that since lower consumption of UPFs lead to higher intake of unprocessed ones, the nutritional contribution of the latter remains preeminent compared to the former. Another hypothesis relies on the fact that individuals reporting higher intakes of UPFs (i.e., sweets) may substitute them with selected unprocessed ones with similar taste (i.e., fruits), potentially resulting in only specific nutritional modification of the diet (i.e., decrease in fruit-derived vitamins, such as vitamin C). In contrast, an increasing trend in sodium intake by increased dietary energy share of UPFs was found in the present study. Interestingly, this finding is in line with results reported in another study conducted on the Italian population [37], while was rather not observed in studies exploring dietary nutritional variations by UPF consumption in non-Mediterranean countries. The consistency of this result across the Mediterranean region suggests that a rationale behind it should be explored, despite it is quite unclear why it could not be observed in non-Mediterranean countries. One can hypothesize that the dietary shares of UPFs in such countries (expressed as dietary energy share from UPFs) might be more influenced by foods not rich in sodium, while in Mediterranean countries the relatively small percentage of UPF consumed might be richer in sodium. However, this hypothesis does not match with our findings, as most of the highest contributors of UPFs are sweet foods. Moreover, when looking at previously published data on major food sources of micronutrients in this cohort [55], about 50% of dietary sodium is derived by grains, with a higher contribution from bread, which has been reported to decrease as UPF consumption increase, thus not allowing for an interpretation of the results. Another hypothesis may suggest that among the most consumed UPFs have been reported fast foods (intended as ultra-processed meats), which are typically overloaded with sodium both to improve the taste and durability of the products [56]. As there is a huge discrepancy across quintiles of UPF consumption in fast food consumptions, ranging from 1.1% to 7.5% of energy share, it may be hypothesized that most of the sodium intake related to UPF consumption is related to their share in fast foods. A final conclusion cannot be drafted from the observed data and further studies are needed to investigate whether dietary sodium intake would, in fact, increase whether a rise in UPF consumption is observed in the Italian population. Consistently, when comparing the average consumption of UPFs in individuals by rate of compliance with nutritional recommendations, individuals meeting the recommendations for sodium, fiber, total fats, vitamin C, and potassium reported consuming less UPFs, suggesting that in this Southern Italian sample, these were the dietary factors more negatively affected by preference of UPFs over the other categories of NOVA food groups.

Irrespective of the relation between dietary energy share of UPFs and compliance with nutritional recommendations examined, there is agreement in the scientific literature (including the results in the present study) that individuals with higher adherence to the Mediterranean diet tend to consume less UPFs [18,37,57]. It is noteworthy to emphasize that the energy shares of various food groups across the NOVA categories by level of adherence to the Mediterranean diet displayed in this study shows that the percentage from groups NOVA 2 culinary processed and NOVA 3 processed did not substantially changed across categories of adherence to the Mediterranean diet while the main differences relied on an alternate consumption of NOVA 1 unprocessed/minimally processed at expenses of NOVA 4 UPFs with increasing adherence to the Mediterranean diet. Albeit it may seem rather obvious that increased consumption of foods characterizing this dietary pattern may lead to higher intake of fruit and vegetables, whole grains, legumes, and olive oil, accompanied with lower consumption of meat, a traditional Mediterranean diet as could be observed in southern Italian islands may also reflect a higher availability of fresh and locally produced foods and a preference for home cooking as dictated by a long-standing cultural heritage. However, results from this study also depicted a background profile of individuals consuming higher shares of UPFs characterized by younger age, smokers, average physically active and often eating out, reflecting a modern active lifestyle. Thus, the changes in behaviors toward more stressful and work-based lifestyles, the rise in availability of UPFs which provide the commodity for out-of-home eating and ready-to-eat foods in a context where time for conviviality is limited, is slowing shaping a new scenario in several Mediterranean countries and might, in the near future, impact also more remote locations, such as Mediterranean islands. Importantly, from the additional analyses we conducted by age groups, we reported that although the absolute average intake of UPFs was higher among younger individuals, the extreme consumption was actually not higher; moreover, we also found that the consumption of certain unprocessed foods was higher in younger than in older participants in the lowest category of UPF consumption. These observations suggest that the contribution of UPFs to the dietary patterns in younger and older adults may substantially differ: on one side, younger individuals consume more UPFs (possibly as a result of their lifestyle) with a large variety of foods, including unprocessed ones; on the other side, older individuals may consume less UPFs, but those with higher intake also tend to have a lower consumption of unprocessed foods and a less varied diet. Assuming that the associations between UPF consumption and higher risk of obesity and other various health issues reported in the scientific literature are convincing, it is important to monitor the consumption of UPFs in all age groups and populations, especially those with traditionally low consumption, to predict potential rise in non-communicable diseases.

The present study is important to provide further information regarding the consumption of UPFs in the Italian population, particularly individuals living in Sicily (in which a higher adherence to traditional dietary patterns compared to northern regions is generally registered). Compared to previous studies, we also provided in-depth analysis for diet quality testing for UPF consumption by various a priori defined dietary patterns and putting in relation UPF consumption with meeting national and international nutritional guidelines. However, potential limitations of this study should be considered when interpreting the results. First, the FFQ was not specifically designed to capture the level of processing of its items; although we followed a procedure widely used in other cohort studies REF, we cannot rule out the possibility of an under-estimation of UPF intake failing to identify the consumption of specific food items not listed or identified in the FFQ. However, certain UPF groups are rather not or poorly consumed in the population (i.e., frozen foods), thus we may hypothesize that the findings represent a reliable proxy of UPF consumption in this sample. In addition, this study relies on self-reported dietary data, which may suffer from recall bias and over/underestimation of quantities. Finally, the NOVA classification aims to provide a proxy of the level of food processing, rather than being based on unequivocal, distinct physical/chemical aspects of foods, thus it may not entirely capture the expected exposure.

## 5. Conclusions

In conclusion, consumption of UPF in southern Italy is in line with those reported in some other Mediterranean countries, with preferences toward certain unprocessed or minimally processed foods (such as fresh vegetables, legumes, and pasta) that somehow may displace the rise in interest for UPFs. As for the relation with diet quality, increase in UPF consumption negatively impacted the nutrient profile, although with a lesser extent compared to other studies. Notably, different patterns of UPF consumption have been registered for younger and older individuals. Considering the current scenario, it is important to monitor the intake of UPFs in the general population and provide targeted education programs by age groups to inform on what are UPFs before their availability and popularity put the grounds on general dietary habits.

## Figures and Tables

**Table 1:** Mean absolute and relative daily energy intake according to NOVA food groups in the study sample (*n =* 1936).

NOVA Food Groups	Kcal	% of Total Energy Intake
*Unprocessed or minimally processed foods*		
Red meat and poultry	73.2	3.0
Fish and sea foods	90.8	3.6
Milk and unprocessed dairy	69.0	2.8
Eggs	3.8	0.2
Grains and pasta	249.1	9.9
Fruits	162.5	6.8
Vegetables	84.4	3.4
Potatoes	16.0	0.6
Nuts	81.1	3.3
Legumes	124.8	4.6
*Processed culinary ingredients*		
Vegetable oils	74.0	3.0
Animal fats	7.1	0.3
Table sugar	14.7	0.6
Fruit juice (natural)	11.4	0.5
*Processed foods*		
Breads	387.1	15.9
Cheese	104.8	4.2
Beer, wine and liquors	60.9	2.5
Processed meats (cured)	30.0	1.2
*Ultra-processed foods*		
Fast foods	87.8	3.4
Ultra-processed dairy	32.1	1.3
Breakfast cereals	19.4	0.8
Biscuits, pastries, cakes	79.8	3.3
Confectionery and creams	38.3	1.5
Ice creams	65.8	2.7
Salty snacks	20.3	0.7
Carbonated soft-drinks	15.8	0.6
Margarine	4.2	0.1
Alcoholic-distilled drinks	8.0	0.3
Confectioned juices	14.4	0.6

**Table 2:** Percentage of energy shares of NOVA food groups across quintiles of the daily energy share of UPFs in the study sample (*n =* 1936).

	Quintiles of the Dietary Contribution of UPFs (% of Total Dietary Energy)						
NOVA Food Groups (% of Total Dietary Energy)	Q1	Q2	Q3	Q4	Q5	*p*-Value	*p* for Trend
*Unprocessed or minimally processed foods*	43.0	40.1	40.9	37.5	31.6	<0.001	<0.001
Red meat and poultry	2.9	3.0	3.0	2.8	3.1	0.243	0.661
Fish and seafoods	3.5	3.3	3.5	3.6	3.9	0.317	0.114
Milk and unprocessed dairy	2.8	3.2	2.9	2.8	2.5	0.082	0.062
Eggs	0.1	0.2	0.1	0.1	0.2	<0.001	0.257
Grains and pasta	9.8	11.0	10.9	9.5	8.4	<0.001	0.135
Fruits	7.9	7.3	6.9	5.8	5.8	<0.001	<0.001
Vegetables	4.1	3.2	3.1	3.1	3.3	<0.001	0.063
Potatoes	0.5	0.5	0.7	0.7	0.6	0.003	0.004
Nuts	2.6	2.7	3.4	3.6	4.1	<0.001	<0.001
Legumes	6.0	4.4	4.6	3.7	4.3	<0.001	0.053
*Processed culinary ingredients*	6.2	6.0	5.7	5.6	4.9	<0.001	<0.001
Plant oils	3.3	3.2	3.1	3.0	2.6	<0.001	<0.001
Animal fats	0.2	0.2	0.3	0.3	0.4	0.001	<0.001
Table sugar	0.5	0.6	0.6	0.7	0.7	<0.001	<0.001
Fruit juice (natural)	0.5	0.5	0.5	0.4	0.5	0.174	0.138
*Processed foods*	44.6	42.3	37.6	35.9	30.5	<0.001	<0.001
Breads	20.4	19.3	15.7	13.2	11.0	<0.001	<0.001
Cheese	3.8	4.3	4.3	4.1	4.8	0.002	0.001
Beer, wine and liquors	2.9	2.1	2.2	2.6	2.7	0.014	0.862
Processed meats (cured)	0.9	1.1	1.3	1.4	1.5	<0.001	<0.001
*Ultra-processed foods*	6.3	12.0	16.3	21.6	34.2	<0.001	<0.001
Fast foods	1.1	1.8	2.4	4.2	7.5	<0.001	<0.001
Ultra-processed dairy	0.8	1.1	1.3	1.4	1.9	<0.001	<0.001
Breakfast cereals	0.1	0.7	1.0	1.1	0.9	<0.001	<0.001
Biscuits, pastries, cakes	1.1	2.4	2.8	3.4	6.8	<0.001	<0.001
Confectionery and creams	0.5	0.9	1.3	1.5	3.4	<0.001	<0.001
Ice creams	0.8	1.5	2.4	3.2	5.8	<0.001	<0.001
Salty snacks	0.2	0.3	0.5	0.6	2.1	<0.001	<0.001
Carbonated soft-drinks	0.1	0.3	0.7	0.7	1.4	<0.001	<0.001
Margarine	0.0	0.1	0.1	0.2	0.3	<0.001	<0.001
Alcoholic-distilled drinks	0.1	0.2	0.3	0.4	0.6	<0.001	<0.001
Confectioned juices	0.1	0.3	0.5	0.6	1.3	<0.001	<0.001

**Table 3:** Mean total energy intake and nutrient content of the overall diet according to quintiles of the energy share of UPFs in the study sample (*n =* 1936).

	Quintiles of the Dietary Contribution of Ultra-Processed Foods (% of Total Dietary Energy)						
Nutrients	Q1	Q2	Q3	Q4	Q5	*p*-Value	*P* For Trend
Energy (kcal)	2019.0 (717.3)	2072.7 (703.4)	2093.4 (733.3)	2035.4 (713.6)	2360.4 (1118.1)	<0.001	<0.001
Energy (KJ)	8168.6 (2980.3)	8393.3 (2894.7)	8499.9 (3052.4)	8259.7 (2947.1)	9589.0 (4638.2)	<0.001	<0.001
Protein (g/d)	87.3 (40.1)	87.4 (31.0)	87.6 (32.6)	84.9 (32.2)	95.5 (52.4)	0.002	0.033
Lipids (g/d)	50.9 (18.4)	56.7 (18.3)	60.7 (21.5)	63.5 (24.9)	81.6 (41.0)	<0.001	<0.001
Cholesterol (mg/d)	163.8 (100.8)	182.9 (80.5)	189.9 (86.1)	203.0 (110.4)	262.4 (145.5)	<0.001	<0.001
Saturated fatty acids (%)	19.1 (7.3)	22.2 (8.3)	23.6 (8.6)	24.8 (9.2)	33.3 (16.6)	<0.001	<0.001
MUFA (%)	22.6 (7.0)	24.5 (7.6)	25.9 (9.4)	26.8 (11.1)	32.3 (15.6)	<0.001	<0.001
PUFA (%)	10.4 (4.4)	10.8 (4.0)	11.5 (5.8)	11.3 (5.3)	13.6 (8.5)	<0.001	<0.001
Total n-3 PUFA (%)	1.7 (0.8)	1.7 (0.8)	1.7 (0.7)	1.7 (1.0)	1.8 (1.1)	0.873	0.393
Seafood n-3 PUFA (%)	0.6 (0.6)	0.6 (0.5)	0.5 (0.4)	0.6 (0.7)	0.5 (0.7)	0.866	0.710
Plant n-3 PUFA (%)	1.1 (0.5)	1.2 (0.5)	1.2 (0.5)	1.2 (0.5)	1.2 (0.7)	0.197	0.075
Carbohydrates (g/d)	309.4 (126.2)	314.2 (127.9)	309.8 (121.7)	288.4 (104.6)	319.8 (153.8)	0.009	0.753
Total fiber (g/d)	38.5 (24.3)	34.5 (15.6)	33.3 (14.7)	29.6 (13.2)	31.4 (20.6)	<0.001	<0.001
Sodium (mg/d)	2713.2 (1065.1)	2899.2 (1203.7)	2890.6 (987.9)	2967.4 (1098.9)	3192.5 (1582.0)	<0.001	<0.001
Potassium (mg/d)	4104.0 (2327.0)	3800.0 (1443.7)	3820.1 (1595.1)	3593.0 (1410.5)	3984.2 (2360.1)	0.002	0.125
Vitamin A (retinol eq.)	953.2 (579.9)	905.7 (463.4)	905.6 (411.5)	843.6 (393.4)	884.2 (496.5)	0.027	0.008
Vitamin C (mg/d)	195.9 (158.7)	171.8 (111.4)	163.9 (96.3)	146.7 (83.2)	155.0 (122.4)	<0.001	<0.001
Vitamin E (mg/d)	8.7 (3.6)	8.7 (3.2)	9.0 (3.5)	8.9 (4.0)	10.0 (5.7)	<0.001	<0.001
Vitamin D (microg/d)	5.3 (5.0)	5.4 (4.7)	5.7 (5.6)	5.9 (7.2)	6.9 (9.5)	0.010	0.001
Vitamin B12 (microg/d)	6.5 (12.0)	6.1(3.8)	6.6(4.5)	7.1(5.9)	8.3(7.3)	<0.001	<0.001

## Data Availability

The data that support the findings of this study are available upon reasonable request.

## Supplementary Materials

The following supporting information can be downloaded at: https://www.mdpi.com/article/10.3390/ijerph191811360/s1. Table S1: Percent distribution and standard deviations of food group consumption by level of processing across background variables. *P*-values indicate statistical differences in ultra-processed food (UPF) consumption between groups. Table S2: Percentage of energy shares of NOVA food groups across quintiles of the daily energy share of UPFs by age groups. Table S3: Mean (and standard deviation) total energy intake and nutrient content of the overall diet according to quintiles of the energy share of UPFs by age groups. Table S4: Percentage (and standard deviations) of UPF consumption in individuals meeting and not meeting the European and Italian nutritional recommendations for selected macro- and micro-nutrients. Table S5: Percent distribution and standard deviations of food group consumption by level of processing across level of adherence to selected a-priori defined healthy dietary patterns. *P*-values indicate statistical differences in ultra-processed food (UPF) consumption between groups.
