# Supplementary material for: Ultra-Processed Food Consumption and Relation with Diet Quality and Mediterranean Diet in Southern Italy
Source: Int J Environ Res Public Health. 2022 Sep 9;19(18):11360. doi: 10.3390/ijerph191811360 (PMC9517140; doi:10.3390/ijerph191811360)
Supplement: Supplementary file 1 [file ijerph-19-11360-s001.zip › ijerph-1881558-supplementary.pdf]

Supplementary Table S1. Percent distribution and standard deviations of food group consumption by level of processing across background variables. P-values indicate statistical differences in ultra-processed food (UPF) consumption between groups.

	Unprocessed/minimally processed foods	Processed culinary ingredients	Processed foods	UPFs	*P-value*
			mean % (SD)		
**Sex**					0.674
Men	37.8 (13.0)	5.6 (2.7)	38.5 (13.7)	18.1 (10.0)	
Women	38.3 (12.4)	5.7 (2.7)	38.1 (12.9)	17.9 (10.2)	
**Age groups**					<0.001
<35 y	36.2 (12.5)	5.3 (2.6)	37.1 (12.7)	21.3 (9.9)	
35-49 y	39.4 (13.0)	5.4 (2.7)	36.5 (13.6)	18.7 (9.8)	
50-65 y	40.2 (12.5)	5.3 (2.7)	38.1 (12.3)	16.3 (9.4)	
>65 y	37.2 (12.2)	6.1 (2.6)	42.1 (13.5)	14.6 (10.2)	
**Marital status**					<0.001
Unmarried/widowed	36.6 (13.0)	5.6 (2.6)	38.5 (13.1)	19.5 (10.7)	
Married	39.5 (12.4)	5.5 (2.8)	38.1 (13.3)	16.9 (9.6)	
**Educational level**					<0.001
Low	36.5 (12.5)	5.7 (2.8)	41.1 (13.0)	16.6 (10.3)	
Medium	40.1 (13.1)	5.4 (2.5)	35.8 (13.3)	18.7 (10.2)	
High	38.3 (11.9)	5.7 (2.7)	37.4 (12.7)	18.6 (9.5)	
**Occupational level**					0.076
Unemployed	36.1 (12.2)	5.7 (2.7)	39.5 (13.2)	18.6 (11.2)	
Low	37.5 (12.3)	5.8 (2.6)	40.0 (12.5)	16.7 (10.1)	
Medium	37.3 (12.3)	6.1 (2.6)	39.0 (12.8)	17.5 (10.2)	
High	37.0 (11.8)	5.4 (2.7)	40.2 (12.5)	17.4 (9.2)	
**Smoking status**					0.046
Never	39.3 (12.7)	5.5 (2.7)	37.5 (13.2)	17.7 (9.5)	
Current	37.7 (12.8)	5.2 (2.5)	38.2 (13.1)	19.0 (11.5)	
Former	35.3 (11.5)	6.0 (2.7)	41.4 (13-0)	17.4 (10.3)	
**Physical activity level**					<0.001
Low	39.3 (13.5)	5.6 (3.1)	38.3 (13.6)	16.8 (10.5)	
Medium	36.7 (12.2)	5.5 (2.7)	38.7 (12.4)	19.1 (10.0)	
High	40.9 (12.9)	5.4 (2.7)	36.5 (13.3)	17.2 (9.2)	
**Breakfast habits**					0.579
Never/seldom	39.5 (12.8)	5.7 (2.8)	36.8 (13.0)	18.2 (10.1)	
Often/always	38.2 (12.6)	5.6 (2.7)	38.3 (13.2)	17.9 (10.1)	
**Snacking habits**					0.001
Never/seldom	38.0 (12.2)	6.0 (2.8)	40.4 (12.8)	15.6 (9.9)	
Often/always	38.5 (12.8)	5.6 (2.7)	37.2 (13.2)	18.7 (10.1)	
**Out-of-home eating**					<0.001
Never/seldom	38.5 (12.9)	6.0 (2.9)	39.3 (13.6)	16.2 (10.6)	
Often/always	38.0 (12.4)	5.1 (2.4)	37.1 (12.7)	19.7 (9.3)	

Supplementary Table S2. Percentage of energy shares of NOVA food groups across quintiles of the daily energy share of UPFs by age groups.

	**Quintiles of the dietary contribution of ultra-processed foods (% of total dietary energy)**							
	**<35 y**		**35-49 y**		**50-65 y**		**>65 y**	
**NOVA food groups (% of total dietary energy)**	**Q1**	**Q5**	**Q1**	**Q5**	**Q1**	**Q5**	**Q1**	**Q5**
Unprocessed or minimally processed foods	46.8	31.4**	44.9	33.5**	43.6	30.7**	40.2	29.0**
Red meat and poultry	3.1	3.1	2.9	3.0	2.9	3.4	2.9	2.7
Fish and seafoods	4.2	3.0*	3.9	5.1*	3.7	4.8	3.0	3.2
Milk and unprocessed dairy	3.7	2.7	2.3	2.9	2.4	1.8*	3.2	2.0*
Eggs	0.1	0.1	0.1	0.2*	0.1	0.2*	0.1	0.2*
Grains and pasta	10.0	9.3	9.2	7.7**	9.9	8.7*	10.1	7.1**
Fruits	10.8	5.9**	8.4	6.0*	8.7	5.2**	6.9	5.2
Vegetables	5.9	3.1**	4.7	4.2*	3.9	2.8*	3.3	2.7*
Potatoes	0.6	0.6	0.5	0.6*	0.6	0.7	0.4	0.7*
Nuts	2.6	3.0	3.1	6.8**	3.4	2.9	1.7	3.3*
Legumes	7.7	4.2*	7.5	5.6**	4.7	2.9*	5.4	4.2*
Processed culinary ingredients	5.6	5.2	6.2	4.8*	6.0	4.4*	6.4	4.7*
Plant oils	3.1	2.6*	3.2	2.8*	3.3	2.2**	3.3	2.8*

Animal fats	0.2	0.3	0.2	0.5*	0.1	0.3*	0.2	0.3
Table sugar	0.4	0.8**	0.5	0.5	0.3	0.7**	0.6	0.5*
Fruit juice (natural)	0.5	0.5	0.6	0.3	0.8	0.7*	0.2	0.3
Processed foods	41.5	31.1**	42.1	27.9**	43.8	32.3**	47.8	31.6**
Breads	19.6	9.1**	19.1	11.3**	19.8	13.5*	21.8	12.8**
Cheese	3.8	5.3**	3.5	4.4*	4.1	4.5	3.8	4.0*
Beer, wine and liquors	1.9	2.0	3.6	3.0	2.8	3.0	2.9	3.9
Processed meats (cured)	1.3	1.3	1.0	1.4*	1.0	1.8**	0.6	1.6**
Ultra-processed foods	6.2	33.3**	6.8	34.7**	6.6	34.1**	5.7	35.8**
Fast foods	1.4	7.6**	1.3	8.1**	0.9	7.3**	0.9	6.3**
Ultra-processed dairy	0.7	1.6**	0.7	2.6**	1.1	1.4*	0.6	1.7**
Breakfast cereals	0.1	0.9*	0.1	1.0**	0.1	0.8**	0.1	0.7**
Biscuits, pastries, cakes	0.8	5.6**	1.2	7.9**	1.2	6.4**	1.0	8.9**
Confectionery and creams	0.6	2.5**	0.5	4.3**	0.7	4.7**	0.4	3.1**
Ice creams	0.8	5.4**	0.7	6.5**	0.8	4.6**	0.7	7.0**
Salty snacks	0.2	2.0**	0.2	2.3**	0.2	2.1**	0.1	2.1**
Carbonated soft-drinks	0.2	1.6**	0.2	1.2**	0.1	1.4**	0.1	1.1**
Margarine	0.0	0.3	0.0	0.5*	0.0	0.1*	0.0	0.1
Alcoholic-distilled drinks	0.1	0.3	0.3	0.6*	0.1	1.0**	0.1	0.5*

Confectioned juices	0.2	1.3*	0.1	1.1*	0.1	1.8**	0.1	1.0**

* denotes *P* <0.05, ** denotes *P* <0.001 as statistically significant difference between all quintiles of UPF intake.

Supplementary Table S3. Mean (and standard deviation) total energy intake and nutrient content of the overall diet according to quintiles of the energy share of UPFs by age groups.

	Quintiles of the dietary contribution of ultra-processed foods (% of total dietary energy)							
	<35 y		35-49 y		50-65 y		>65 y	
**NOVA food groups**	**Q1**	**Q5**	**Q1**	**Q5**	**Q1**	**Q5**	**Q1**	**Q5**
Energy (kcal)	2200.6 (1030.6)	2229.4 (1083.1)	2075.8 (868.1)	2590.0 (1380.0)**	2039.3 (580.6)	2323.2 (919.9)	1911.0 (560.3)	2330.3 (745.6)**
Energy (KJ)	8896.7 (4350.2)	9037.5 (4456.3)	8420.6 (3602.3)	10573.7 (5753.4)**	8237.5 (2421.2)	9397.0 (3807.7)	7729.4 (2289.2)	9470.9 (3131.2)**
Protein (g/d)	100.4 (58.1)	91.2 (47.6)*	92.3 (57.3)	105.7 (67.7)	86.2 (25.0)	95.7 (45.6)	81.0 (26.0)	86.9 (32.4)
Lipids (g/d)	55.6 (23.6)	76.8 (39.4)**	52.2 (23.5)	90.9 (52.3)**	52.8 (15.1)	79.3 (32.1)**	47.1 (14.3)	79.9 (23.5)**
Cholesterol (mg/d)	179.0 (128.1)	241.5 (120.7)**	174.7 (152.7)	294.5 (192.0)**	163.2 (67.3)	272.3 (140.9)**	152.7 (64.2)	245.7 (87.0)**
Saturated fatty acids (%)	20.2 (8.2)	31.3 (15.8)**	18.7 (8.5)	36.9 (20.8)**	20.0 (7.0)	38.8 (14.2)**	18.4 (6.4)	32.7 (9.5)**
MUFA (%)	24.4 (9.2)	30.4 (14.9)**	22.6 (8.0)	35.8 (19.7)**	23.5 (6.1)	31.4 (12.7)**	21.3 (5.8)	31.8 (2.3)**
PUFA (%)	11.7 (5.9)	12.7 (8.4)*	11.0 (6.0)	14.9 (10.5)**	10.5 (3.3)	13.3 (6.1)*	9.5 (3.2)	13.6 (6.0)**
Total n-3 PUFA (%)	1.9 (1.1)	1.2 (0.7)	1.6 (0.7)	1.8 (1.2)*	1.8 (0.8)	2.0 (1.3)	1.6 (0.7)	1.9 (1.1)

Seafood n-3 PUFA (%)	0.7 (0.9)	0.4 (0.3)*	0.4 (0.4)	0.6 (0.7)	0.6 (0.6)	0.8 (1.1)	0.5 (0.5)	0.5 (0.5)
Plant n-3 PUFA (%)	1.2 (0.5)	1.2 (0.7)	1.1 (0.5)	1.2 (0.7)*	1.2 (0.4)	1.2 (0.5)	1.0 (0.4)	1.3 (1.0)*
Carbohydrates (g/d)	338.9 (181.5)	305.8 (152.0)*	312.6 (145.2)	345.0 (176.4)*	312.1 (108.9)	313.2 (135.9)	296.0 (101.7)	318.2 (126.3)
Total fiber (g/d)	47.5 (45.3)	29.9 (19.4)**	41.8 (30.3)	35.7 (26.1)*	38.7 (13.6)	28.2 (14.4)**	33.4 (13.6)	31.2 (16.3)*
Sodium (mg/d)	2951.8 (1198.3)	3241.3 (1700.3)	2824.9 (1181.4)	3176.6 (1487.6)	2772.3 (1082.0)	3302.0 (1685.9)*	2523.0 (899.5)	2932.7 (1235.4)*
Potassium (mg/d)	5013.8 (4019.7)	3759.9 (2043.3)**	4493.8 (3161.8)	4571.7 (3159.6)*	4215.0 (1343.3)	3783.6 (1798.0)	3489.2 (1128.9)	3709.0 (1734.7)
Vitamin A (retinol eq.)	1196.0 (1008.1)	860.5 (464.5)**	967.0 (625.4)	962.6 (507.9)	1064.6 (483.2)	856.4 (559.2)*	784.8 (343.8)	829.8 (475.2)
Vitamin C (mg/d)	254.8 (272.3)	146.5 (102.5)**	219.2 (202.3)	173.9 (143.4)*	216.6 (118.2)	160.3 (143.0)**	147.3 (69.9)	134.8 (95.7)
Vitamin E (mg/d)	10.2 (5.5)	9.4 (5.1)	9.1 (4.5)	11.5 (7.1)**	8.9 (2.5)	9.4 (4.6)	7.8 (2.3)	9.7 (4.7)*
Vitamin D (mg/d)	6.0 (6.2)	5.2 (7.0)	5.2 (5.9)	8.9 (12.1)*	5.8 (5.2)	8.5 (11.6)*	4.8 (3.7)	5.4 (4.7)
Vitamin B12 (mg/d)	8.3 (17.3)	7.5 (5.3)	9.1 (21.1)	9.6 (8.6)	5.6 (5.2)	9.1 (9.0)**	5.0 (2.4)	7.1 (6.6)*

* denotes *P* <0.05, ** denotes *P* <0.001 as statistically significant difference between all quintiles of UPF intake.

Supplementary Table S4. Percentage (and standard deviations) of UPF consumption in individuals meeting and not meeting the European and Italian nutritional recommendations for selected macro- and micronutrients.

	**EFSA recommendations**			**LARN recommendations**		
	Not meeting	***Meeting***	***P-value***	***Not meeting***	***Meeting***	***P-value***
Carbohydrates	15.3 (8.2)	20.1 (10.9)	<0.001	15.3 (8.2)	20.1 (10.3)	<0.001
Protein	18.8 (11.7)	17.7 (9.7)	0.068	18.6 (11.4)	17.7 (9.7)	0.118
Fat	18.4 (10.1)	12.5 (8.0)	<0.001	18.4 (10.1)	12.5 (8.0)	<0.001
Fiber	20.7 (11.1)	16.6 (9.4)	<0.001	22.0 (10.8)	15.2 (8.6)	<0.001
Sodium	19.2 (10.0)	13.0 (9.5)	<0.001	18.3 (9.9)	16.5 (11.0)	0.002
Potassium	18.6 (10.4)	17.3 (9.8)	0.007	18.2 (10.0)	17.5 (10.3)	0.174
Vitamin A	18.4 (10.4)	17.6 (10.0)	0.079	18.8 (10.7)	17.5 (9.8)	0.009
Vitamin B12	15.4 (11.0)	18.8 (9.7)	<0.001	16.0 (12.6)	18.0 (10.0)	0.107
Vitamin C	20.4 (11.0)	17.0 (9.6)	<0.001	20.7 (11.2)	17.1 (9.6)	<0.001
Vitamin D	17.8 (10.1)	20.8 (10.2)	0.003	17.8 (10.1)	20.8 (10.2)	0.003
Vitamin E	17.4 (9.7)	19.7 (11.4)	<0.001	12.3 (9.6)	20.7 (11.7)	<0.001

Supplementary Table S5. Percent distribution and standard deviations of food group consumption by level of processing across level of adherence to selected a-priori defined healthy dietary patterns. P-values indicate statistical differences in ultra-processed food (UPF) consumption between groups.

	Unprocessed/ minimally processed foods	Processed culinary ingredients	Processed foods	UPFs	*P-value*
		mean % (SD)			<0.001
**Mediterranean diet**					
Low	36.2 (12.2)	5.9 (2.8)	38.6 (12.3)	19.8 (10.4)	
Medium	40.9 (12.8)	5.5 (2.6)	38.1 (14.6)	16.0 (9.5)	
High	43.9 (11.4)	5.2 (2.3)	37.2 (12.8)	14.7 (8.4)	
**DASH diet**					<0.001
Low	32.6 (11.8)	6.3 (3.1)	39.3 (13.5)	22.4 (11.9)	
Medium	39.1 (12.4)	5.7 (2.6)	37.4 (12.8)	18.3 (10.0)	
High	41.9 (12.5)	5.3 (2.5)	39.5 (13.9)	14.1 (7.5)	
**A-HEI**					<0.001
Low	33.7 (12.6)	6.1 (2.8)	40.9 (12.8)	19.8 (10.8)	
Medium	38.6 (11.5)	5.7 (2.4)	38.7 (13.3)	17.6 (10.0)	
High	43.7 (11.9)	5.3 (2.8)	35.2 (12.9)	16.4 (9.1)	
**DQI-I**					<0.001
Low	33.0 (12.0)	5.9 (3.0)	37.2 (13.2)	24.5 (10.8)	
Medium	38.9 (11.6)	6.0 (2.8)	38.2 (12.3)	17.6 (9.2)	
High	42.6 (12.6)	5.2 (2.2)	39.1 (14.0)	13.6 (7.7)	
